# Small-Molecule Inhibition of MuRF1 Prevents Early Disuse-Induced Diaphragmatic Dysfunction and Atrophy

**DOI:** 10.3390/ijms24043637

**Published:** 2023-02-11

**Authors:** Fernando Ribeiro, Paula K. N. Alves, Luiz R. G. Bechara, Julio C. B. Ferreira, Siegfried Labeit, Anselmo S. Moriscot

**Affiliations:** 1 Department of Anatomy, Institute of Biomedical Sciences, University of São Paulo, São Paulo 05508-000, Brazil; 2 DZHK Partner Site Mannheim-Heidelberg, Medical Faculty Mannheim, University of Heidelberg, 68169 Mannheim, Germany; 3 Myomedix GmbH, 69151 Neckargemünd, Germany

**Keywords:** diaphragm, mechanical unloading, contractile dysfunction, atrophy, MuRF1, MuRF2

## Abstract

In clinical conditions such as diaphragm paralysis or mechanical ventilation, disuse-induced diaphragmatic dysfunction (DIDD) is a condition that poses a threat to life. MuRF1 is a key E3-ligase involved in regulating skeletal muscle mass, function, and metabolism, which contributes to the onset of DIDD. We investigated if the small-molecule mediated inhibition of MuRF1 activity (MyoMed-205) protects against early DIDD after 12 h of unilateral diaphragm denervation. Wistar rats were used in this study to determine the compound’s acute toxicity and optimal dosage. For potential DIDD treatment efficacy, diaphragm contractile function and fiber cross-sectional area (CSA) were evaluated. Western blotting investigated potential mechanisms underlying MyoMed-205’s effects in early DIDD. Our results indicate 50 mg/kg bw MyoMed-205 as a suitable dosage to prevent early diaphragmatic contractile dysfunction and atrophy following 12 h of denervation without detectable signs of acute toxicity. Mechanistically, treatment did not affect disuse-induced oxidative stress (4-HNE) increase, whereas phosphorylation of (ser632) HDAC4 was normalized. MyoMed-205 also mitigated FoxO1 activation, inhibited MuRF2, and increased phospho (ser473) Akt protein levels. These findings may suggest that MuRF1 activity significantly contributes to early DIDD pathophysiology. Novel strategies targeting MuRF1 (e.g., MyoMed-205) have potential therapeutic applications for treating early DIDD.

## 1. Introduction

Disuse-induced diaphragmatic dysfunction (DIDD) is characterized by the early and progressive loss of contractile function and atrophy in response to mechanical unloading. Relevant causes of DIDD include phrenic nerve injury, leading to unilateral or bilateral diaphragm denervation [1,2], and mechanical ventilation (MV) commonly used for treating critically ill patients in intensive care units (ICUs) [3]. Ventilator-induced diaphragmatic dysfunction (VIDD) has been the most studied sub-type of DIDD due to its high clinical relevance, supported by accumulating evidence during the COVID-19 pandemic [4,5]. In this context, DIDD can promote exercise intolerance, reduced quality of life, higher risk of complications, weaning failure, hospital length of stay, mortality, and healthcare-associated costs [6,7,8].

The early onset of diaphragmatic contractile dysfunction and atrophy characterizes DIDD. In rodent models, even brief periods (e.g., 12–72 h) of MV significantly reduce the diaphragm fiber’s contractile function and cross-sectional area (CSA) [9,10]. Similar levels of diaphragmatic force loss are also observed in the first days of unilateral diaphragm denervation (UDD) in rats [11,12]. Furthermore, both UDD and MV experimental models result in a rapid and preferential loss of sarcomeric proteins, particularly the myosin heavy chain (MHC) isoforms [9,10,11,12]. Similarly, human studies confirm the early and progressive nature of disuse-induced diaphragm weakness and atrophy using different approaches (e.g., clinical tests, ultrasound, and biopsies) [13,14,15,16]. Even so, the cellular and molecular signaling pathways involved in the onset of DIDD remain poorly understood.

Disuse-induced diaphragmatic dysfunction and atrophy is a multifactorial disorder. Disuse rapidly downregulates the IGF1-Akt-mTOR pathway [17,18,19], induces mitochondrial dysfunction [20,21], oxidative stress [18,22], and upregulates key proteolytic pathways, including Ca^2+^-dependent proteases (e.g., calpains), the autophagic lysosomal and the ubiquitin-proteasome systems (UPS) in the diaphragm [22,23,24]. In DIDD, the UPS plays a key role through the rapid activation of the so-called atrogenes, MAFbx (Atrogin-1) and MuRF1, increasing muscle protein ubiquitination and causing functional changes or degradation by the proteasome [18,22,25,26]. Despite this, the role of UPS in the early development of DIDD requires further investigation. In this regard, selective proteasome activity inhibitors seem to attenuate, but not prevent, diaphragm dysfunction and atrophy induced by MV in rats. These studies also indicate that despite the proteasome activity inhibition, the activation of atrogenes (MAFbx and MuRF1) and the levels of poly-ubiquitylated proteins remained elevated [26,27]. In contrast, efficacy interventions against early DIDD, such as calpain inhibition [28], HSP72 overexpression, or exercise [29], also significantly suppress the atrogenes. Additionally, in a MuRF1 knockout (KO) model, Hooijman and co-workers [30] observed that MuRF1 deletion fully protected against early loss of diaphragm contractile force following MV. These findings may suggest that MuRF1 plays a crucial role in early DIDD pathophysiology.

The model used by Hooijman and co-workers [30] is a complete MuRF1 KO inactivating this gene in all tissues and at all developmental stages (see also [31]). In this study, we tested if MuRF1 is mechanistically involved in early DIDD by treating animals with a novel small-molecule targeting MuRF1 activity (MyoMed-205), concomitantly with the induction of DIDD. Herein, we hypothesized that inhibition of MuRF1 activity can impact the early development of DIDD driven by mechanical unloading. Rats subjected to 12 h of diaphragm disuse induced by UDD were treated with either a vehicle (placebo) or MyoMed-205 (MuRF1 inhibitor), immediately after denervation, and diaphragm functional, structural, and molecular analysis was performed and compared to Sham control animals. Compared to vehicle-treated and control animals, the small-molecule-mediated inhibition of MuRF1 activity prevented both diaphragm fiber contractile dysfunction and atrophy following 12 h of disuse. Mechanistically, MyoMed-205’s protective effect was associated with phospho-serine 473 Akt activation, downregulation of FoxO1, inhibition of phospho (ser632) HDAC4, and MuRF2 expression. Targeting of MuRF1 as a potential strategy to treat disuse-induced diaphragmatic dysfunction and atrophy should be further evaluated, such as in animal models that mimic critical illness and ICU conditions.

## 2. Results

### 2.1. MyoMed-205 Acute Toxicological Study

Our first objective was to determine the acute toxicity of Myomed-205 in rats. We chose this method because the experimental model used in this study also includes investigating the acute effects of this molecule on diaphragm function and mass in the early DIDD model. For this, rats received a single dosage of 300 or 2000 mg/kg bw of MyoMed-205. Then, the animals were examined daily over 14 days for signs of toxicity, including behavioral and clinical observations, ranging from mild features such as visual changes to more severe features such as tremors or death (Table 1).

**Figure 1:**
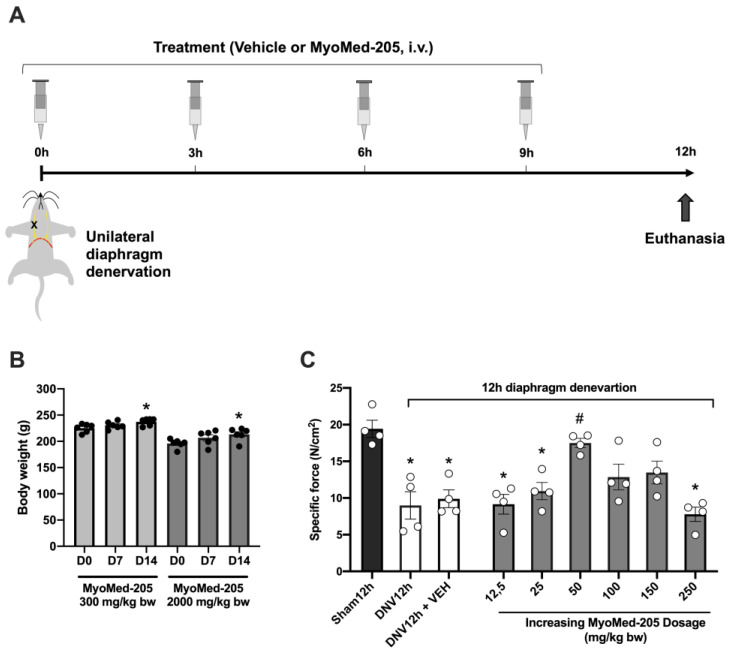
MyoMed-205 compound acute toxicity and dose–response study in animals. (**A**) Schematic illustration of MyoMed-205 dose–response study design in a DIDD experimental model induced by 12 h of unilateral diaphragm denervation. (**B**) Body weight development of rats in the acute toxicological study of the MyoMed-205 compound (D0 = day 0; D7 = day 7; and D14 = day 14). (**C**) MyoMed-205 dose–response effect upon diaphragmatic contractile dysfunction after 12 h of unilateral diaphragm denervation. Data are shown as mean ± standard error of the mean (* *p* < 0.05 vs. Sham12h, ^#^ *p* < 0.05 vs. DNV12h + VEH, *n* = 4–6).

During the observation period, no signs of toxicity in the rats administered with the 300 mg/kg bw of the compound were noticed. Similarly, despite administering the highest dose (2000 mg/kg bw), as suggested by Organization for Economic Co-Operation and Development, OECD, protocol (for details, see Materials and Methods), no signs of toxicity were observed based on the characteristics daily assessed. Regarding organ macroscopy, neither 300 mg/kg bw nor 2000 mg/kg bw resulted in any indications of swelling, shape, color, or weight changes (Table 2). Finally, neither dose of Myomed-205 impacted the animal’s body weight growth over time (Figure 1B). Therefore, the MyoMed-205 compound was classified as Category 5 (unclassified) according to the GHS (Globally Harmonized Classification System for Chemical Substances and Mixtures). This compound’s acute oral LD50 value cut-off was estimated to be 5000 mg/kg bw for female rats.

### 2.2. MyoMed-205 Dose–Response Curve in a DIDD Experimental Model

Next, we determined the optimal dosage of Myomed-205 by assessing the diaphragm contractile force. For this purpose, we intravenously administered increasing dosages of Myomed-205 (12.5 to 250 mg/kg bw) to rats subjected to 12 h of diaphragm denervation. Then, a muscle strip was isolated for the in vitro contractility assessment. In either denervated rats or denervated rats treated with vehicle, a drop of approximately 50 percent was observed in the diaphragm’s maximal tetanic force (Figure 1C). Regarding the dose–response curve, the 12.5 and 25 mg/kg bw dosages did not protect against diaphragm force loss after 12 h of denervation. In contrast, the 50 mg/kg bw dosage was able to significantly prevent diaphragmatic contractile dysfunction. Higher dosages (e.g., 100 and 150 mg/kg bw) mitigated diaphragm force loss. In the highest dose (250 mg/kg bw) used, impaired diaphragm force was also noted (Figure 1C).

### 2.3. MuRF1 Inhibition Prevents Early Disuse-Induced Diaphragmatic Contractile Dysfunction and Atrophy after 12 h of Denervation

**Figure 2:**
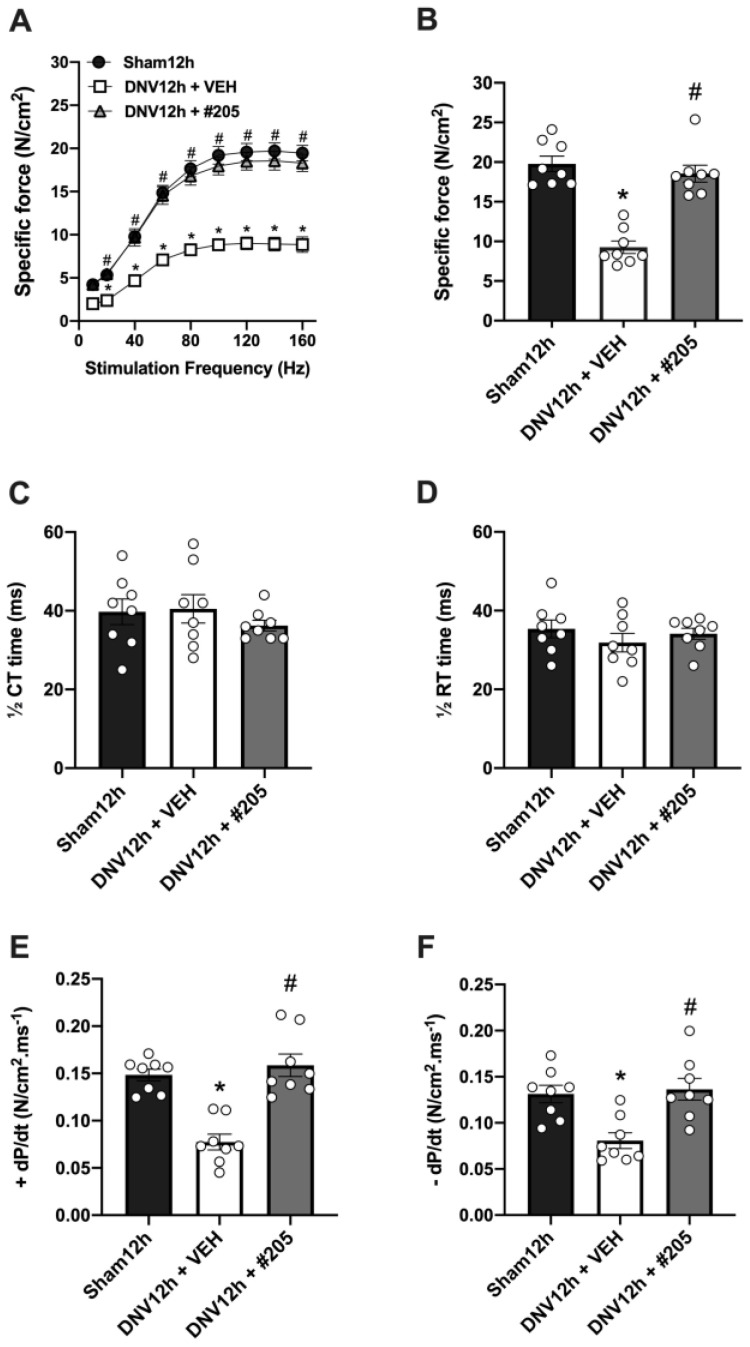
MyoMed-205 prevents early disuse-induced diaphragmatic contractile dysfunction after 12 h of denervation. (**A**) Force–frequency curve. (**B**) Maximal tetanic force. (**C**) Half-time for contraction (1/2 CT). (**D**) Half-time for relaxation (1/2 RT). (**E**) Rate of force development during contraction (+dP/dt). (**F**) Rate of force development during relaxation (−dP/dt). Data are shown as mean ± standard error of the mean (* *p* < 0.05 vs. Sham12h, ^#^ *p* < 0.05 vs. DNV12h + VEH, *n* = 8).

After determining the compound’s optimal dosage for this experimental setup, we further evaluated the diaphragm contractile function in rats treated with the Myomed-205 at 50 mg/kg bw. As expected, the denervated group treated with vehicle showed decreased diaphragmatic contractile force at almost all stimulation frequencies tested (20–160 Hz, see Figure 2A). In contrast, the Myomed-205 optimal dosage prevented diaphragm force loss at both force–frequency and maximal tetanic force assessments (Figure 2A,B). Neither the half-time for contraction (1/2 CT) (Figure 2C) nor the half-time for relaxation (1/2 RT) (Figure 2D) changed among groups. However, both rates of force development during contraction (+dP/dt) and relaxation (−dP/dt) were impaired in denervated rats treated with vehicle, but normalized by Myomed-205 treatment (Figure 2E,F).

**Figure 3:**
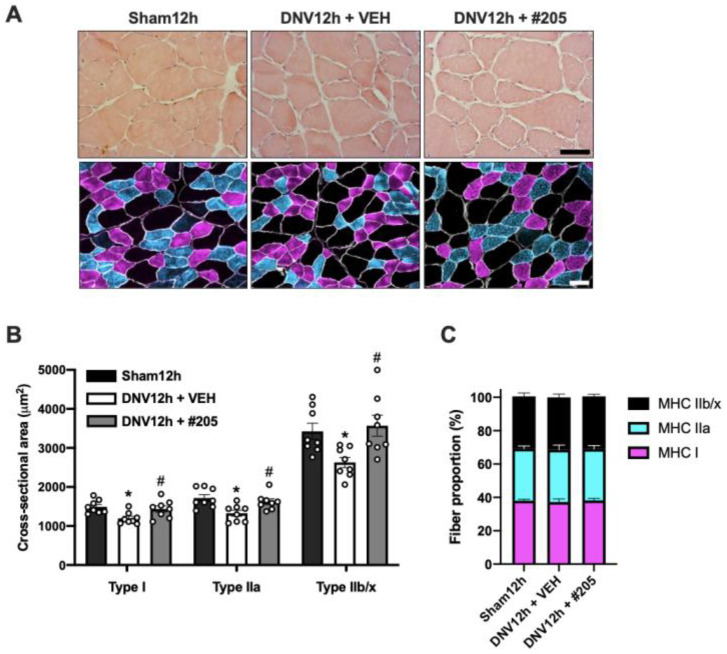
MyoMed-205 prevents early disuse-induced diaphragmatic fiber atrophy after 12 h of denervation. (**A**) Representative photomicrographs of the costal diaphragm muscle stained with hematoxylin and eosin (HE) and using immunofluorescence anti-MHC isoforms I (violet), IIa (light blue), IIb/x (black), and dystrophin (white). Scale bar = 50 μm. (**B**) Mean of diaphragm fiber types (I, IIa, and IIb/x) cross-sectional area (CSA, μm^2^). (**C**) Fiber type distribution in the diaphragm. Data are shown as mean ± standard error of the mean (* *p* < 0.05 vs. Sham12h, ^#^ *p* < 0.05 vs. DNV12h + VEH, *n* = 8).

Histomorphometric analysis was employed to determine whether the protective effect of MyoMed-205 treatment on early contractile dysfunction could be linked to morphological changes. The histological analysis of the diaphragm, stained with HE, revealed conserved gross structural aspects among groups, including muscle cells containing typical polygonal shape, peripherally located nuclei, and no signs of injury or inflammatory-like cell infiltrate (Figure 3A). Morphometric measurements of the immunolabeled diaphragm for MHC isoforms (Figure 3A) showed that 12 h denervation significantly decreased the CSA of all fiber types: type I (19%), type IIa (22%), and type IIb/x (23%), compared with Sham12h group. Interestingly, MyoMed-205 treatment significantly prevented diaphragm fiber atrophy (Figure 3B). No changes were observed in diaphragm fiber type distribution among groups (Figure 3C).

### 2.4. MuRF1 Inhibition Affects HDAC4, FoxO1, MuRF2 and Akt, but Not Oxidative Stress, in the Early DIDD

**Figure 4:**
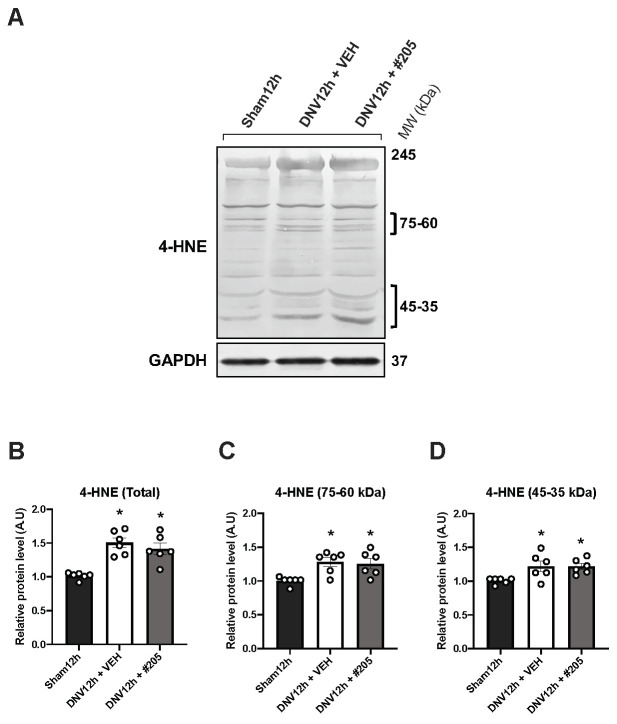
Western blotting analysis of the 4-hydroxynonenal (4-HNE) oxidative stress marker in diaphragm after 12 h of denervation. (**A**) 4-HNE Western blot representative lanes (MW = molecular weight). (**B**) Densitometry of total 4-HNE considering the 180–30 kDa range. (**C**) Densitometry of 4-HNE in the specific 75–60 kDa range. (**D**) Densitometry of 4-HNE in the specific 45–35 kDa range. Data are shown as mean ± standard error of the mean (* *p* < 0.05 vs. Sham12h, ^#^ *p* < 0.05 vs. DNV12h + VEH, *n* = 6).

To gain insight into the mechanisms underlying the protective effect of Myomed-205 upon diaphragm structure and function in an early disuse condition, we measured levels of proteins containing the lipid peroxidation product 4-HNE (4-hydroxynonenal) as a marker for oxidative stress (Figure 4A). 4-HNE-protein adducts within the 180–30 kDa molecular weight range increased approximately 50% in the diaphragm after 12 h of denervation (Figure 4B). The Myomed-205 treatment did not affect this (Figure 4B). Similarly, within the most responsive specific ranges of 75–60 kDa and 45–35 kDa, denervation increased levels, while Myomed-205 had no effect (Figure 4C,D).

**Figure 5:**
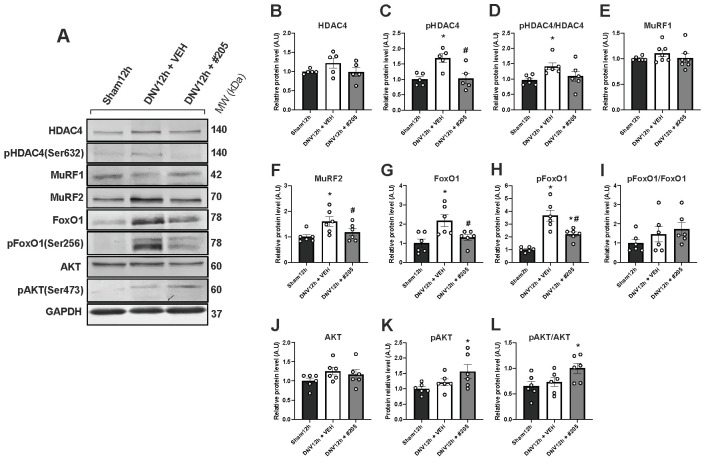
Western blotting analysis of proteins involved in the regulation of skeletal muscle mass and function. (**A**) Representative bands of each protein assessed by Western blot (MW = molecular weight). (**B**–**D**) Densitometry of total HDAC4, phospho (Ser632) HDAC4, and the ratio of phospho/total HDAC4 protein levels. (**E**) Densitometry of MuRF1 protein levels. (**F**) Densitometry of MuRF2 protein levels. (**G**–**I**) Densitometry of total FoxO1, phospho (Ser256) FoxO1, and the ratio of phospho/total FoxO1 protein levels. (**J**–**L**) Densitometry of total Akt, phospho (Ser473) Akt, and the ratio of phospho/total Akt protein levels. Data are shown as mean ± standard error of the mean (* *p* < 0.05 vs. Sham12h, ^#^ *p* < 0.05 vs. DNV12h + VEH, *n* = 5–7).

Next, we determined whether skeletal muscle function and mass regulatory signaling pathways might be responsive to Myomed-205’s treatment in the early DIDD model (Figure 5A). HDAC4 total levels were unaffected after 12 h of denervation and Myomed-205 treatment also had no impact (Figure 5B). In contrast, phosphorylated (Ser632) HDAC4 was elevated in the diaphragm of denervated animals treated with vehicle, but not in those treated with Myomed-205 (Figure 5C). The proportion of phosphorylated HDAC4 increased in denervated animals, but this effect was blocked by Myomed-205 (Figure 5D). MuRF1 protein levels were unaffected by either denervation or Myomed-205 (Figure 5E), whereas MuRF2 levels were increased by 60% after 12 h of denervation and was blocked by Myomed-205 treatment (Figure 5F). Denervated animals had dramatically elevated total FoxO1 levels, whereas Myomed-205 effectively downregulated FoxO1 expression (Figure 5G). Similarly, denervation significantly increased phosphorylated (Ser 256) FoxO1, and Myomed-205 treatment mitigated this response (Figure 5H). Due to the simultaneous increase in total and phosphorylated FoxO1 levels, no significant changes were observed in phospho/total FoxO1 ratio among groups (Figure 5I). Analysis of the anabolic factor Akt revealed stable levels among groups (Figure 5J). However, the MyoMed-205 increased phosphorylated (Ser473) Akt levels (Figure 5K), leading to an increased phospho/total Akt ratio (Figure 5L).

## 3. Discussion

Mechanical unloading induces early and progressive loss of diaphragmatic fiber’s contractile function and atrophy. Likewise, the clinical effects of disuse-induced diaphragm dysfunction include exercise intolerance, diminished quality of life, an increased risk of morbidity, mortality and healthcare costs. In this study, we demonstrated that MyoMed-205, a novel small-molecule inhibitor of MuRF1, prevents early diaphragmatic dysfunction and atrophy caused by the UDD model. This model deprives one diaphragm dome of all contractile function due to a lack of neural system electrical stimulation. The denervated diaphragm is only stretched passively by the active diaphragm on the opposite side [32]. Within hours of mechanical unloading, proteolytic signaling pathways are activated in the diaphragm, resulting in a significant loss of fiber contractile force and mass [9,10,11,12]. The unilateral diaphragm denervation model is at least as severe as the MV model in promoting diaphragm disuse, but less complicated to manage than the prolonged ventilation model’s multiple invasive procedures and multidrug environment, which could potentially interact with MyoMed-205. Although the MV model mimics clinical conditions present in ICUs, where disuse-induced diaphragmatic weakness and wasting are prevalent complications, we decided to examine the effect of MyoMed-205 in DIDD using the UDD model for the sake of simplicity and lower interference from a multidrug context. Denervation resulted in an approximately 50% reduction in the diaphragm specific force after 12 h, which is comparable to previous reports [11,12], validating the successful induction of diaphragm paralysis. In addition, disuse decreased the CSA of all diaphragm fiber types (MHC I (19%), MHC IIa (22%), and MHC IIb/x (23%)), similar to the diaphragm’s atrophy degree observed in other DIDD experimental studies [18,28,29]. Future research employing the MV model is warranted given that our results in this DIDD model indicate a solid diaphragm’s functional and structural protection.

The initial objective of this study was to determine the optimal MyoMed-205 dosage in the context of early DIDD. In order to accomplish this, we conducted an acute toxicity study to determine the impact of this molecule’s potential acute side effects. This acute toxicity study was conducted under the 2001 OECD Guideline for Testing of Chemicals, Section 4: Health Effects, 423. Initially, an arbitrary single dose of 300 mg/kg bw was administered, and the animals were observed daily for 14 days. As they exhibited no signs of toxicity (Table 1 and Table 2), another group of animals was administered the maximum dose recommended by the OECD protocol (2000 mg/kg bw), and no behavioral, clinical, or gross organ damage was observed. In addition, there were no impairment in animal’s body weight gain over two weeks, indicating that the test item was well tolerated and safe for acute use. MyoMed-205 was classified as Category 5 (unclassified) by the GHS (Globally Harmonized System of Classification and Labeling of Chemical Substances and Mixtures), and the acute oral LD50 value cut-off for female rats was estimated to be 5000 mg/kg bw. These data indicate that there are no signs of acute toxicity. Future studies will need to examine organ histology for potential signs of tissue damage.

Next, we investigated the dose–response effect of MyoMed-205 on diaphragm contractile dysfunction following 12 h of unilateral denervation. The observed loss of diaphragm tetanic force was comparable between the solely denervated and denervated treated with vehicle groups, indicating that the vehicle solution (DMSO-PEG400-Saline 0.9%) did not interfere with diaphragm contractility (Figure 1C). This result corroborates with a previous report, which verified that a DMSO-based vehicle did not alter the diaphragm’s contractile properties in the MV experimental model [26]. Thus, a series of relatively low doses (relative to the OECD protocol) were tested when given with DMSO-PEG400-Saline. We observed that 50 mg/kg bw of Myomed-205 promoted the most effective protection against DIDD. Higher dosages, such as 100 mg/kg bw and 150 mg/kg bw, provided partial protection against diaphragm force loss. In addition, the highest dose (250 mg/kg bw) had no protective effect against DIDD. These findings indicate that MyoMed-205 has a restricted window of high-efficacy dosage to treat DIDD. Based on the findings of the oral acute toxicity study presented herein (summarized in Table 1 and Table 2), this small range of action is likely unrelated to toxic effects, according to our understanding. Therefore, the less effective dosages (100 and 150 mg/kg bw) and the ineffective dosage (250 mg/kg bw) likely activate or inhibit specific pathways that ultimately inhibit the compound’s protective effect. MuRF1 plays a crucial role not only in the regulation of skeletal muscle mass and function, but also in muscle energy metabolism, including glucose management [33,34,35,36], so systemic effects must also be taken into account. To the best of our knowledge, this is the first time a dose–response analysis has been performed on Myomed-205, and we anticipate that investigating more specific dosage windows (e.g., 25 to 100 mg/kg bw) including other functional and biochemical parameters will aid in gaining further insight into the optimal dosage of MyoMed-205 and, clarify whether the functional rescue of the compound occurs in a dose–dependent manner. In addition, when advancing this inhibitor into pre-clinical or clinical settings, the limited range of high-efficacy dosage should be taken into account.

In order to further test and analyze the MyoMed-205 protective effect, we assessed diaphragm contractile properties and fibers CSA in a subsequent independent experiment. When analyzing the sparing effect of MyoMed-205 on diaphragm contractile function capacity in greater detail, we observed that the maximal specific force of the diaphragm in the denervated treated with vehicle animals decreased by about half, which is in line with our initial dose–response screening experiment. The lack of a difference between groups in the half contraction and half relaxation times may suggest that calcium handling is not impaired at this time point in denervated animals treated either with the vehicle or the MyoMed-205. Notably, the CSA of all fiber types analyzed (type I, type IIa, and type IIb/x) in the denervated vehicle group decreases approximately by 20% compared to the denervated treated with MyoMed-205 and Sham groups. Therefore, the robust loss of diaphragm force with preserved contractility is not attributable solely to the diaphragm fiber’s atrophy. One possible explanation could involve the rapid damage and disruptive effect that mechanical unloading has on the sarcomeric structure of the diaphragm. Oxidative stress has been identified as an early activating mechanism that contributes to the functional impairment of contractile proteins by promoting Ca^2+^ desensitization, protease activation, and sarcomeric damage and degradation [22]. Accordingly, we investigated whether an explanation for the protective effect of MyoMed-205 on early DIDD could involve the reduction of oxygen species production. Attempts have been made, with varying degrees of success, to reduce the accumulation of oxygen-containing species in decommissioned diaphragms by employing antioxidants such as vitamin E analogs or N-acetylcysteine [18,37]. In the MV model, the authors were able to mitigate disuse-induced diaphragm force loss and atrophy. We have evaluated the levels of 4-HNE as a marker of oxidative stress. Although we observed an increase in 4-HNE levels after 12 h denervation, MyoMed-205 had no effect on oxidative stress. This evidence suggests that MuRF1 inhibition does not affect the disuse-induced production of oxygen species in the diaphragm. 

Next, we examined the expression of two E3-ligases that are targeted by Myomed-205, e.g., MuRF1 and MuRF2. Both E3 ligases regulate sarcomeric proteins. We did not detect any significant changes in MuRF1 protein levels following 12 h of denervation. We should emphasize that the effects of rat unilateral denervation models upon MuRF1 expression are still lacking in the literature; nonetheless, evidence from rat ventilation models suggests a strong elevation in MuRF1 protein levels only after longer ventilation periods (≥5 days) [38,39]. Nevertheless, disuse-induced upregulation of MuRF1 activity in the diaphragm possibly occurs earlier than its protein expression. In fact, previous studies have shown that as soon as 6–12 h of disuse can increase the levels of ubiquitinated proteins [40,41], known to be influenced by MuRF1 activity, accompanied by increased proteasome activity [26,40,42,43]. This also could be in line with the preferential loss of myofibrillar proteins including contractile elements targeted by MuRF1 [26,38,44]. In contrast, we observed that disuse rapidly increases MuRF2 protein expression in comparison to the control. Myomed-205 had no significant effect on MuRF1 protein levels; however, it effectively repressed MuRF2 expression after 12 h denervation. MuRF1 has historically been linked to the degradation of sarcomeric proteins (e.g., titin, nebulin, the nebulin-related protein NRAP, troponin-I, troponin-T, myotilin, T-cap, MyBP-C, MyLC1, MyLC2, and MHC) [45,46,47]. In addition, Baehr and co-workers 2021 [35] recently suggests that MuRF1 regulates 169 lysines on 56 proteins, including contractile proteins of both thick and thin myofilament, as well as proteins involved in metabolism or other proteolytic systems (e.g., autophagy). Also, previous research indicates that MuRF1 and MuRF2 share at least 35 targets, such as the recognition of specific Z-disk components, transcriptional regulators, translational machinery, and mitochondrial metabolism [31,46]. Recent animal studies have demonstrated that either MuRF1 gene deletion (knockout model) [48] or the small-molecule mediated inhibition of its activity [49] downregulate MuRF2 protein levels. Consequently, it is conceivable that MyoMed-205’s mediated suppression of the MuRF1 close relative MuRF2 may also be involved in the protective effect observed in MyoMed-205 treated animals. 

It is also conceivable that this MuRF1 inhibitor could also affect other pathways involved in the regulation of skeletal muscle mass or function. For example, HDAC-4 is involved in the mechanisms underlying Myomed-205 protection from DIDD. HDAC4 is a histone deacetylase involved in the regulation of muscle mass and homeostasis [50,51]. The expression of total HDAC4 protein was not altered in any denervated groups. In fact, it was previously reported that early diaphragm disuse, such as 12 h of MV, did not alter HDAC4 gene expression [41]. However, after 12 h of denervation, a strong upregulation of HDAC4 phosphorylation at serine 632 was observed, which was prevented by MyoMed-205 treatment. It has been reported that phosphorylated HDAC4 is exported to the cytosol and deacetylates essential substrates for the regulation of muscle homeostasis, such as MHC, PGC1a, and Hsc70. Inhibition of HDAC4 prevents atrophy by increasing myosin heavy chain and PGC-1a protein levels [50]. Consequently, another potential mechanism involved in the protective effect of MyoMed-205 on early DIDD could involve the prevention of HDAC4-mediated acetylation of these targets.

FoxO1 is another important factor in the diaphragm’s early disuse-induced dysfunction and atrophy. It has been demonstrated that upon mechanical unloading, this transcriptional regulator is rapidly activated in the diaphragm [18,19,41]. In response to 12 h of denervation, we detected a significant increase in total FoxO1 protein expression as well as the form phosphorylated at the serine 256 residue. This simultaneous response of total and phosphorylated forms could be interpreted as a general activation of the system, where absolute levels of phosphorylated FoxO1 are accumulating in the nucleus, while the phosphorylated/total ratio is not significantly altered. Intriguingly, MyoMed-205 strongly inhibits this response, resulting in a decrease in total FoxO1 protein levels and a trend toward an increase in the phosphorylated/total ratio, despite failing to reach statistical significance. This, in our interpretation, represents a deactivation of FoxO1, as its total levels decrease while its relative phosphorylated fraction rises. Due to the fact that MyoMed-205 can increase levels of phosphorylated Akt (serine 473), this phenomenon may be related to the simultaneous activation of Akt. It is well known that activated Akt (Ser473) phosphorylates FoxO1 (Ser256), resulting in its nuclear exit and inactivation [52,53]. Moreover, this finding is in line with the recent findings of Labeit and co-workers [36], who described that MuRF1 KO mice show increased Akt activation through phosphorylation of serine 473 in skeletal muscles, and adenovirus-associated re-expression of MuRF1 in these KO mice normalizes phosphorylated (serine 473) Akt levels.

Overall, the present data indicate that MyoMed-205 effectively preserves the diaphragm fiber’s contractile function and mass. Although the utilized model does not fully encompass clinical settings involving mechanical ventilation, it is a promising starting point that could establish this MuRF1 activity inhibitor as a potential future therapeutic device to combat diaphragm dysfunction and worsening intensive care unit outcomes.

## 4. Materials and Methods

### 4.1. Animals

Twelve young adult female Wistar rats were used in the MyoMed-205 oral acute toxicity study (Phase 1). Forty-eight young adult male Wistar rats were used for the MyoMed-205 dose–response and effect study in the DIDD experimental model (Phases 2 and 3). Animals were maintained in standard cages under controlled environmental conditions (24 ± 1 °C, 12 h/12 h light–dark cycle) with access to standard food and water ad libitum. All experimental procedures followed the institutional animal care regulations and the approved protocols CEUA ICB USP (#8728030320).

### 4.2. Experimental Design

Study Phase 1 consisted of MyoMed-205 oral acute toxicology analysis, which was carried out according to the Organization for Economic Co-Operation and Development (OECD) Guideline for Testing of Chemicals—Section 4: Health Effects, 423, 2001. Twelve rats divided into groups of three animals were tested in a four steps process at the dose levels of 300 and 2000 mg/kg bw, as the initial and maximal doses tested, respectively. The volume administered to each animal was calculated according to the body weight determined on the day of treatment. After dosing by gavage, the animals were observed for 14 days to evaluate mortality, behavioral and clinical alterations, and tissue macroscopic changes following euthanasia.

According to the Phase 1 outcomes, Study Phase 2 was designed, consisting of the MyoMed205 dose–response investigation which aimed to determine the compound’s optimal dosage for intravenous treatment in the DIDD experimental model. For this, thirty-six animals were initially randomly divided into 9 groups (*n* = 4/group): 3 control groups (Sham12h; DNV12h and DNV12h + Vehicle) and 6 experimental groups representing increasing dosages of MyoMed-205 (#205): (DNV12h + #205 [12.5 mg/kg bw]; DNV12h + #205 [25 mg/kg bw]; DNV12h + #205 [50 mg/kg bw]; DNV12h + #205 [100 mg/kg bw]; DNV12h + #205 [150 mg/kg bw] and DNV12h + #205 [250 mg/kg bw].

After the dose–response study, Phase 3 involved additional experiments carried out using only MyoMed-205 optimal dosage in order to consolidate the investigation of the effects of the MuRF1 inhibitor upon diaphragm dysfunction and atrophy following 12 h of disuse induced by denervation. For this, further 12 animals were randomly divided into 3 groups (*n* = 4/group): Sham12h; DNV 12h + Vehicle (DNV12h + VEH); and DNV 12h + MyoMed-205 (DNV12h + #205), totalizing 8 animals per group considering phases 2 and 3. After 12 h, animals were euthanized and the right costal diaphragm was collected for functional, morphological and molecular analysis (Figure 1A).

### 4.3. MyoMed-205 Acute Toxicity

As mentioned previously, the acute toxicity analysis was initially conducted with an initial dose of 300 mg/kg bw and the maximal testing dose of 2000 mg/kg bw. This starting dose was based on historical information from studies carried out with the same active ingredient and data from the literature [36,49,54]. The feed supplied to the animals was interrupted at the end of the day previous to the application of the MyoMed-205 compound, which was applied diluted in the concentration of 0.20 g mL^−1^ using corn oil as a vehicle. The volume administered was calculated according to the body weight of each animal determined on the day of treatment. The administration was performed by gavage, using a suitable metal cannula attached to a syringe. Animals were returned to ad libitum feeding 3 h later.

The time interval between treatment groups was determined according to the duration and severity of signs of toxicity. Rat’s body weights were monitored on the day of the administration of the MyoMed-205, Day 0 (D0), and after 7 days (D7) and 14 days (D14). Additionally, these animals were examined for mortality and systemic signs of toxicity, especially during the first 4 h after dosing and 14 days following #205 administration. Observations included but were not limited to: changes in skin and fur, eyes, and mucous membranes alteration in the respiratory, circulatory, autonomic, and central nervous systems, and somatosensorial and motor activity. Furthermore, close attention was applied to the apparition of convulsions, salivation, lethargy, tremors, diarrhea, coma and death. 

At the end of the observation period, all survivor’s animals were weighed and euthanized in a carbon dioxide chamber. A second method of euthanasia (cervical dislocation) was realized to confirm the animal’s death. These animals next were subjected to necropsy. This included a minute examination of the external body surface, all orifices, thorax, pelvic and abdominal cavities, and contends. All the observations were registered.

### 4.4. MyoMed-205 Formulation for Intravenous Administration

The MyoMed-205 compound was solubilized in an 8 mL formulation vehicle consisting of DMSO-PEG400-Saline 0.9% (20%-50%-30%, respective proportions). Working formulation batches were freshly prepared immediately before the in vivo experimental use. For this, MyoMed#205 was first supplemented with the calculated volume of DMSO, vortexed for 1 min, and sonicated at ~40 °C for 2 min. Next, the corresponding volumes of PEG400 and Saline 0.9% were added to the formulation, vortexed for 1 min, and sonicated at ~40 °C for 2 min. Treatment delivery of vehicle (VEH) or MyoMed-205 (#205) solutions were managed into 4 split doses (2 mL/dose) administered each 3 h/3 h intervals over the 12 h disuse protocol period. The animal’s caudal vein was used for the intravenous delivery of both vehicle and MyoMed205 treatments, and the first dose was administered immediately after inducing unilateral diaphragm denervation.

### 4.5. Unilateral Diaphragm Denervation (UDD)

The procedure to induce UDD surgically was previously described in detail [12]. Here, the animals were pre-anesthetized with acepromazine (2.5 mg/kg bw, i.p.) and after 30 min, rats were anesthetized with ketamine (100 mg/kg bw) and xylazine (5 mg/kg bw) administered intraperitoneally. In the surgical plane, the right phrenic nerve was exposed and transected at the lower neck region. The efficacy of UDD induction was verified by the absence of contractile activity of the right diaphragm. Then, the wound was sutured and treated with a topical antiseptic. After 12 h, animals were euthanized under anesthesia by exsanguination and the right costal diaphragm was collected for functional, morphological and molecular analysis.

### 4.6. Contractile Function

The in vitro assessment of diaphragm contractile function was performed as described previously [10,54]. The right costal diaphragm was prepared in a Krebs-Hanseleit buffer solution (120.5 NaCl, 4.8 KCl, 1.2 MgSO_4_, 1.2 NaH_2_PO_4_, 20.4 NaHCO_3_, 1.6 CaCl_2_, 10 D-glucose; in mmol/L at a pH of ~7.4) at room temperature equilibrated with 95% O_2_/5% CO_2_ for contractile measurements. Briefly, a diaphragm muscle bundle connected from rib to central tendon was dissected, attached to silk sutures (5-0) at either end, and mounted vertically in a buffer-filled organ bath (~37 °C) for in vitro contractile function to be assessed using a length-controlled lever system (AVS Projects, São Carlos, Brazil). The muscle bundle was set at optimal length and after a 15 min thermo-equilibration period was stimulated over force–frequency protocol between 15 and 160 Hz (120 V; 500 ms train duration; 1.00 ms pulse width; 3 min interval). The muscle then underwent a maximal tetanic force (Po) protocol consisting of two supra-maximal stimuli 160 Hz (120 V; 500 ms train duration; 1.00 ms pulse width; 3 min interval). Additionally, the Po test also was used to determine other contractility measurements such as half-time of contraction (1/2 CT) and relaxation (1/2 RT), as well as the rate for force development during contraction (+dP/dt) and relaxation (−dP/dt). Force (N) was normalized to muscle cross-sectional area (CSA; cm^2^) by dividing muscle mass (g) by the product of Lo (cm) and estimated muscle density (1.056), which allowed specific force in N/cm^2^ to be calculated.

### 4.7. Histomorphometry

Diaphragm cryosections (8 µm thick) were prepared using a cryostat at −20 °C (Leica CM1850 UV, Wetzlar, Germany). Initially, samples were stained with hematoxylin-eosin (HE) for gross histological examination. Next, diaphragm fiber typing was determined by immunofluorescence assay using the following primary antibodies anti-myosin heavy chain (MHC) isoforms (Developmental Studies Hybridoma Bank, University of Iowa): anti-MHC I (#BA-D5, mouse IgG2b, 1:800), Anti-MHC IIa (#SC-71, mouse IgG1, 1:800); and diaphragm muscle membrane was stained using anti-dystrophin antibody (SC#15376, rabbit IgG, 1:500). Primary antibodies were incubated overnight at 4 °C. The secondary antibodies: DyLight 405 goat anti-mouse IgG2b (Jackson ImmunoResearch, West Grove, PA, USA, #115-475-207, 1:200), Cy2 goat anti-mouse IgG1 (Jackson ImmunoResearch #115-225-205, 1:200), and Cy3 donkey anti-rabbit (Jackson ImmunoResearch #711-165-152, 1:200) were used and incubated for 1 h at 37 °C. Diaphragm fiber types IIb and IIx have not been immunolabeled and appear black. Photomicrographs were acquired using Axio Scope A1 (Carl Zeiss Microscopy GmbH, Göttingen, Germany). Diaphragm fiber type proportion and CSA were measured using ImageJ software (v. 1.45 s, National Institutes of Health, Bethesda, MD, USA). To determine the representative mean CSA, 300 fibers were measured by sample.

### 4.8. Western Blotting

Samples were powdered in a liquid nitrogen-chilled mortar and homogenized in RIPA buffer (1 mM EDTA, pH 7.4, 0.625% sodium deoxycholate, 0.625% nonidet P-40, 6.2 mM sodium phosphate and protease and phosphatase inhibitor cocktail) (Thermo Fisher Scientific, Category Number #78445, Rockford, IL, USA). Homogenates were incubated on ice for 10 min, and centrifuged at 10,000× *g* for 10 min at 4 °C after the supernatant containing the total protein was stored at −80 °C, as described previously [55].

Protein concentration was determined by the Bradford method with bovine serum albumin (BSA) as the standard. Equal amounts of total protein (10–45 μg) were loaded onto an 8–12% polyacrylamide gel (SDS-PAGE) and were separated by electrophoresis (60–120 V for 60–120 min). Proteins were transferred to a polyvinylidene difluoride membrane 0.45 µm (Thermo Fisher Scientific, #88518, Rockford, IL, USA ) in a semi-dry system (20 V for 60–120 min). To verify a homogeneous loading, membranes were stained with Ponceau red solution. After that, membranes were washed and then blocked with 5% BSA in Tris-buffered saline with Tween (0.5 M NaCl, 50 mM Tris-HCl pH 7.4, 0.1% Tween 20) for 1 h at room temperature. Subsequently, membranes were washed (3 rounds of 5 min) with Tris-buffered saline containing 0.1% Tween, followed by overnight incubation at 4 °C with primary antibody. The following primary antibodies were used: rabbit anti-4-HNE (1:1000, Sigma Aldrich, #393207, St. Louis, MO, USA), rabbit anti-HDAC4 (1:1000, Cell Signaling, #7628, Danvers, MA, USA), rabbit anti-phospho(Ser632) HDAC4 (1:1000; Sigma Aldrich, #SAB4300155), rabbit anti-FoxO1 (1:1000, Cell Signaling, #2880), rabbit anti-phospho(Ser256) FoxO1 (1:000, Cell Signaling, #9461), rabbit anti-Akt (1:000, Cell Signaling, #9272), rabbit anti-phosphor(Ser473) Akt (1:000, Cell Signaling, #4058), rabbit anti-MuRF1 (1:1000, Myomedix, Neckargemünd, Germany), rabbit anti-MuRF2 (1:1000, Myomedix), rabbit anti-GAPDH (1:3000; Cell Signaling, #2118), and rabbit anti-α-tubulin (1:1000, Cell Signaling, #2125).

Membranes were washed in TBST (3 rounds of 5 min) to remove primary antibodies and treated with secondary antibody solution (1:30,000, goat anti-rabbit peroxidase Jackson ImmunoResearch, #111035003; incubation for 1 h at room temperature). Then, membranes were washed again in TBST (3 rounds of 5 min). Bound proteins were visualized by the Fusion FX5 XT Vilber Loyurmat imaging system, using 2 min of LuminataTM incubation (Millipore, Burlington, MA, USA, #WBLUF0500). GADPH or α-tubulin protein levels were determined as housekeeping control for normalization.

### 4.9. Statistical Analysis

Statistical analysis was performed using Prism (GraphPad Prism 8.0 Software, downloaded from www.graphpad.com, accessed on 5 May 2020). Unpaired *t*-test was used for comparisons between two groups. One-way ANOVA followed by Tukey’s posttest was employed for comparisons between three or more groups. Two-way ANOVA followed by Tukey’s posttest was used to analyze contractile function analysis. Data are presented as mean ± standard error of the mean. Statistical significance was accepted as *p* < 0.05.

## 5. Conclusions

The small-molecule mediated inhibition of MuRF1 (MyoMed-205) prevents disuse-induced diaphragmatic contractile dysfunction and atrophy in rats subjected to 12 h of unilateral diaphragm denervation. MyoMed-205 protective effects involve the downregulation of atrophy-related factors (HDAC4, FoxO1, and MuRF2) and Akt activation. These findings provide a promising starting point for recognizing this inhibitor of MuRF1 activity as a potential future strategy to counteract early DIDD in patients subjected to phrenic nerve injury or mechanical ventilation.

## Figures and Tables

**Table 1:** Behavioral and clinical alterations observed during the experimental period.

**Dose** **(mg.Kg^−1^ bw)**	**Step**	**Animal #**					**observation day**													
			**0**				**1**	**2**	**3**	**4**	**5**	**6**	**7**	**8**	**9**	**10**	**11**	**12**	**13**	**14**
			0:30 h *	10:20 am	12:00 pm	02:00 pm														
300	1	1	0	0	0	0	0	0	0	0	0	0	0	0	0	0	0	0	0	0
		2	0	0	0	0	0	0	0	0	0	0	0	0	0	0	0	0	0	0
		3	0	0	0	0	0	0	0	0	0	0	0	0	0	0	0	0	0	0
**Dose** **(mg.Kg^−1^ bw)**	**Step**	**Animal #**					**observation day**													
			**0**				**1**	**2**	**3**	**4**	**5**	**6**	**7**	**8**	**9**	**10**	**11**	**12**	**13**	**14**
			0:30 h *	09:30 am	11:55 am	12:50 pm														
300	2	1	0	0	0	0	0	0	0	0	0	0	0	0	0	0	0	0	0	0
		2	0	0	0	0	0	0	0	0	0	0	0	0	0	0	0	0	0	0
		3	0	0	0	0	0	0	0	0	0	0	0	0	0	0	0	0	0	0
**Dose** **(mg.Kg^−1^ bw)**	**Step**	**Animal #**					**observation day**													
			**0**				**1**	**2**	**3**	**4**	**5**	**6**	**7**	**8**	**9**	**10**	**11**	**12**	**13**	**14**
			0:30 h *	09:30 am	11:00 am	2:00 pm														
2000	3	1	0	0	0	0	0	0	0	0	0	0	0	0	0	0	0	0	0	0
		2	0	0	0	0	0	0	0	0	0	0	0	0	0	0	0	0	0	0
		3	0	0	0	0	0	0	0	0	0	0	0	0	0	0	0	0	0	0
**Dose** **(mg.Kg^−1^ bw)**	**Step**	**Animal #**					**observation day**													
			**0**				**1**	**2**	**3**	**4**	**5**	**6**	**7**	**8**	**9**	**10**	**11**	**12**	**13**	**14**
			0:30 h *	10:00 am	11:03 am	12:50 pm														
2000	4	1	0	0	0	0	0	0	0	0	0	0	0	0	0	0	0	0	0	0
		2	0	0	0	0	0	0	0	0	0	0	0	0	0	0	0	0	0	0
		3	0	0	0	0	0	0	0	0	0	0	0	0	0	0	0	0	0	0

Legend: 0. visual alterations observed; 1. Skin, pile and eyes alterations; 2. Mucous membranes alterations; 3. Respiratory system alteration; 4. System circulation alteration; 5. Nervous system alteration; 6. Behavior pattern alteration; 7. Convulsions; 8. Salivation; 9. Diarrhea; 10. Lethargy; 11. Tremble; 12. Death. *. After application of Test Item.

**Table 2:** Pathological findings in animals at doses of 300 and 2000 (mg/kg bw).

Treatment	Dose (mg.Kg^−1^)	Animal #	Macroscopic Alterations										
			Skin	Brain	Eyes	Lungs	Heart	Liver	Spleen	Urinary System	G.I.T	R.T	Carcass
1st	300	1	0	0	0	0	0	0	0	0	0	0	0
		2	0	0	0	0	0	0	0	0	0	0	0
		3	0	0	0	0	0	0	0	0	0	0	0
2nd	300	1	0	0	0	0	0	0	0	0	0	0	0
		2	0	0	0	0	0	0	0	0	0	0	0
		3	0	0	0	0	0	0	0	0	0	0	0
3rd	2000	1	0	0	0	0	0	0	0	0	0	0	0
		2	0	0	0	0	0	0	0	0	0	0	0
		3	0	0	0	0	0	0	0	0	0	0	0
4th	2000	1	0	0	0	0	0	0	0	0	0	0	0
		2	0	0	0	0	0	0	0	0	0	0	0
		3	0	0	0	0	0	0	0	0	0	0	0

Legend: G.I.T—Gastrointestinal tract; R.T—Reproductive tract; 0—Not observed alteration; A—Observed alteration (Tissues gross pathological and microscopic evaluation).

## Data Availability

The data that support the findings of this study are available from the corresponding author (A.S.M.), upon reasonable request.
